# Microbiota-Accessible Boron-Containing Compounds in Complex Regional Pain Syndrome

**DOI:** 10.3390/medicina59111965

**Published:** 2023-11-07

**Authors:** Cristina Elena Biţă, Ion Romulus Scorei, Ananu Florentin Vreju, Anca Emanuela Muşetescu, George Dan Mogoşanu, Andrei Biţă, Venera Cristina Dinescu, Ştefan Cristian Dinescu, Cristina Criveanu, Andreea Lili Bărbulescu, Alesandra Florescu, Paulina Lucia Ciurea

**Affiliations:** 1 Department of Rheumatology, University of Medicine and Pharmacy of Craiova, 2 Petru Rareş Street, 200349 Craiova, Romania; cristina.gofita@umfcv.ro (C.E.B.); florentin.vreju@umfcv.ro (A.F.V.); anca.musetescu@umfcv.ro (A.E.M.); stefan.dinescu@umfcv.ro (Ş.C.D.); cristina.criveanu@umfcv.ro (C.C.); andreea.barbulescu@umfcv.ro (A.L.B.); alesandra.florescu@umfcv.ro (A.F.); paulina.ciurea@umfcv.ro (P.L.C.); 2 Department of Biochemistry, BioBoron Research Institute, S.C. Natural Research S.R.L., 31B Dunării Street, 207465 Podari, Romania; 3 Department of Pharmacognosy & Phytotherapy, Faculty of Pharmacy, University of Medicine and Pharmacy of Craiova, 2 Petru Rareş Street, 200349 Craiova, Romania; george.mogosanu@umfcv.ro (G.D.M.); andreibita@gmail.com (A.B.); 4 Department of Health Promotion and Occupational Medicine, University of Medicine and Pharmacy of Craiova, 2 Petru Rareş Street, 200349 Craiova, Romania; venera.dinescu@umfcv.ro

**Keywords:** complex regional pain syndrome, microbiota, boron-containing compounds, prebiotic boron, gut–brain axis, gut–musculoskeletal axis, gut–immune system axis

## Abstract

The microbiota–gut–brain axis has garnered increasing attention in recent years for its role in various health conditions, including neuroinflammatory disorders like complex regional pain syndrome (CRPS). CRPS is a debilitating condition characterized by chronic neuropathic pain, and its etiology and pathophysiology remain elusive. Emerging research suggests that alterations in the gut microbiota composition and function could play a significant role in CRPS development and progression. Our paper explores the implications of microbiota in CRPS and the potential therapeutic role of boron (B). Studies have demonstrated that individuals with CRPS often exhibit dysbiosis, with imbalances in beneficial and pathogenic gut bacteria. Dysbiosis can lead to increased gut permeability and systemic inflammation, contributing to the chronic pain experienced in CRPS. B, an essential trace element, has shown promise in modulating the gut microbiome positively and exerting anti-inflammatory effects. Recent preclinical and clinical studies suggest that B supplementation may alleviate neuropathic pain and improve CRPS symptoms by restoring microbiota balance and reducing inflammation. Our review highlights the complex interplay between microbiota, inflammation, and neuropathic pain in CRPS and underscores the potential of B as a novel therapeutic approach to target the microbiota–gut–brain axis, offering hope for improved management of this challenging condition.

## 1. Introduction

Complex regional pain syndrome (CRPS), also known as reflex sympathetic dystrophy or causalgia, is a debilitating and persistent neuroinflammatory disorder that typically emerges in the aftermath of stressful events, such as surgery or trauma. CRPS can manifest in two forms: CRPS-I affects individuals without confirmed nerve injury, while CRPS-II affects those with associated nerve damage [1]. Both types of CRPS are diagnoses reached by ruling out other potential causes, and they involve pain that is disproportionate to the initial injury, accompanied by the presence of signs in at least two and symptoms in at least three of the following categories: sensory, vasomotor, sudomotor, and motor or trophic [2].

CRPS patients commonly experience decreased mobility, an inability to work, a diminished quality of life (QoL), as well as depression, anxiety, and long-term reliance on opioids [3]. Therefore, it is crucial to provide an early diagnosis and initiate treatment promptly to curb disease progression and enhance patients’ QoL. Conservative approaches, such as medication use, physical therapy interventions, or injections, are among the available treatment options for CRPS. While there is ongoing debate regarding this matter, early symptoms of CRPS are widely believed to be influenced by sympathetic activity, resulting in the adoption of sympathetic blocks for symptom management purposes [4]. Experimental treatments like intravenous immunoglobulin have yielded unsatisfactory results thus far. Tragically, 20–50% of individuals with less than one year’s worth of symptoms do not respond well enough to conservative strategies and may consequently necessitate chronic opioid use or implantation procedures involving neuromodulation devices like spinal cord stimulators or dorsal root ganglion stimulators [5]. As CRPS progresses into its chronic stage, observed efficacy from established treatment methods diminishes even further [2].

The lack of satisfactory treatment for CRPS is due to the incomplete understanding of its root causes. Despite years of research, there is still only a partial understanding of how exactly CRPS develops. The condition involves an abnormal reaction of tissues to injury as well as enhanced sensitivity in both peripheral and central nervous systems (CNS), accompanied by inflammation and autonomic dysfunction. Furthermore, it is thought that genetic factors and psychological influences play a role in the progression of CRPS. Studies have primarily concentrated on the human leukocyte antigen system due to significant alterations in gene expression within this system [1].

CRPS is defined as an immune reaction that promotes inflammations and impairs neuropeptide signaling [6]. This activation of the innate immune system leads to the proliferation of keratinocytes and the release of proinflammatory cytokines, such as interleukin (IL)-6, IL-1, and tumor necrosis factor-alpha (TNF-α) [7]. These cytokines initiate a cascade within the immune system, causing histamine-induced vasodilation, which results in redness, swelling, pain, and warmth during the acute phase of CRPS [8]. Moreover, these proinflammatory cytokines stimulate osteoblasts and osteoclasts, resulting in accelerated bone remodeling and the distinctive osteoporotic alterations observed in chronic CRPS cases [9,10]. Additionally, neuropathic inflammation is thought to be a key contributor to the onset of CRPS. The activation of peripheral nociceptors situated on C-fibers leads to the transmission of pain signals to the dorsal ganglia and the affected tissue [11]. This retrograde transmission produces proinflammatory neuropeptides, such as substance P (SP) and calcitonin gene-related peptide (CGRP) [11].

Currently, there are no known indicators that can be used to predict the likelihood of developing or recovering from CRPS [3]. It is crucial to identify the factors that contribute to recovery to prevent and treat this debilitating condition effectively. We believe that the diversity of symptoms and response to treatment in CRPS may be linked to variations in the gut microbiota. We hypothesize that the gut microbiome (GM) could play a role in both the onset and persistence of CRPS symptoms. Additionally, we suggest that studying the clinical status of individuals affected by CRPS could provide valuable insights into biomarkers related to their microbiota composition, as well as potential therapeutic approaches aimed at preventing long-term pain. We propose exploring how metabolome activity, immune system activation, and microglial activation might be influenced by or connected with changes in the gut microbiota during different stages of CRPS [2].

The correlation between the GM and painful conditions has garnered considerable research interest. Extensive evidence suggests that GM plays a vital role in maintaining human physiological balance, impacting systemic inflammation, immunity, circadian rhythm, and hormone regulation—all of which have been linked to pain. The GM has shown associations with various types of pain, including visceral pain, inflammatory pain, headache, neuropathic pain, chronic pain, and opioid tolerance. The overall genomic material from intestinal microbes has been estimated to be greater than 100 times the size of the human genome. Most gut microbiota bacteria in healthy adults are derived from two phyla, *Firmicutes* and *Bacteroidetes*. These populations are believed to be influenced by various factors at personal, interpersonal, environmental, and geographical levels that shape the composition of the microbiome [12].

Recent research has sparked great interest in the possible involvement of the gut microbiota in pain, as discussed in several well-regarded reviews [13,14]. Animal studies have revealed that a healthy population of gut bacteria is essential for normal perception of visceral pain, while an imbalanced composition can contribute to the development of conditions including irritable bowel syndrome (IBS), neuropathic pain, and inflammatory pain [15,16,17,18]. In human clinical studies, alterations in bacterial diversity or dysbiosis within the GM have been linked to various painful conditions, such as visceral pain, fibromyalgia (FM), and knee arthritis [19,20,21]. It is worth noting that there have been multiple instances where patients with CRPS experienced improvement following the use of antibiotics known to impact the microbiome [22,23]. In these cases, the individuals had been dealing with CRPS for a significant amount of time before being prescribed antibiotics for unrelated reasons. These reports suggest a potential connection between microbiota and long-term management of CRPS [2].

Additionally, in his review titled “Neurogenic neuroinflammation in fibromyalgia and complex regional pain syndrome”, Geoffrey Littlejohn suggests that neuroinflammation may have a “neurogenic” basis, possibly influenced by pain and stress [11]. However, it is premature to solely attribute neuroinflammation and central sensitization to a primary neurogenic origin without considering the well-established presence of coexisting factors, such as small intestinal bacterial overgrowth (SIBO), mitochondrial dysfunction, and vitamin D deficiency associated with gastrointestinal (GI) dysbiosis [24].

Recently, there has been evidence of SIBO in individuals with FM, which refers to the presence of colonic bacteria in the distal small bowel [25]. Furthermore, patients with CRPS have shown impaired intestinal permeability (IP) [26,27,28]. It is suggested that defective immune cell function plays a role in the pathophysiology of both FM and CRPS [29,30,31]. Peripheral blood mononuclear cells from patients with FM and CRPS exhibited reduced messenger ribonucleic acid (mRNA) expression for anti-inflammatory cytokines, indicating an increased inflammatory response [29,30]. It could be hypothesized that a compromised gut barrier may contribute to specific immunological reactions associated with these syndromes. There is evidence suggesting that restoring normal IP could potentially improve disease activity in certain human conditions [32]. Another possible explanation for the elevated IP in both FM and CRPS patients might be related to distress caused by their pain. It is well-established that pain can induce stress, although the level of distress does not necessarily correlate directly with the intensity of pain [32]. Research conducted on animal models and human mucosal investigations supports the role of stress in altering IP [33,34]. One hypothesis could be that pain-related distress independently contributes to changes in IP. Altered IP may arise from various factors, such as SIBO, gut infections, gut inflammation, medication usage, stress levels, trauma, or the use of non-steroidal anti-inflammatory drugs (NSAIDs) [35].

Neuroinflammation plays a role in FM and CRPS as much as GI dysbiosis, vitamin D deficiency, and mitochondrial dysfunction. These independent factors often occur together, and each contributes to the development of both peripheral and central hyperalgesia. The consistent pain relief seen with interventions targeting intestinal dysbiosis (antibiotics), vitamin D deficiency (supplementation), and mitochondrial dysfunction (ubiquinone) demonstrates that these painful conditions have multiple underlying causes that can be addressed [24]. Thus, the timely initiation of therapy is crucial for improving patient prognosis in cases of CRPS, as the symptoms can vary over time. The main goals of treatment are to restore limb functionality, reduce pain, and enhance QoL. This typically involves a multidisciplinary approach that includes patient education, physical and occupational therapy, psychiatric support, and interventions from pain medicine specialists through medications or surgical procedures. To further advance our understanding of this condition’s root causes and develop more targeted therapies, larger-scale clinical studies with higher-quality data are necessary [1].

## 2. Dysbiosis and Degradation of the Mucus Gel Layer in Complex Regional Pain Syndrome

In healthy individuals, the GM maintains a balanced and stable condition characterized by a diverse and abundant microbial population. It is believed that in this state of equilibrium, the GM helps regulate the permeability of the intestinal wall [36,37]. A healthy GM also contributes to the production of essential vitamins and nutrients, some deficiencies of which have been associated with pain [38,39] (e.g., vitamin B complex [40,41,42] and vitamin D [43,44]). When disturbances occur within the GM ecosystem, such as dysbiosis, there can be significant changes to key bacterial species. These alterations may result in modified permeability levels, disruptions in physiological functions, metabolic imbalances, and an increased susceptibility to diseases [12].

One important role of the GM is to support the development and maintenance of a healthy intestinal barrier [45]. This barrier consists of a protective mucosal layer and a monolayer of intestinal epithelial cells that are held together by tight junctions [46,47]. In normal conditions, this barrier prevents microbes and their components from crossing into the underlying immune cells [48]. The presence of beneficial bacteria helps protect the integrity of this barrier by preventing harmful pathogens from colonizing on the surface or invading epithelial cells and entering circulation [49,50]. Additionally, microbial-produced short-chain fatty acids (SCFAs) like butyrate and acetate play a role in preserving the integrity of these tight junction proteins that keep everything in place within our intestines [51,52,53]. Disruption of the epithelial barrier and heightened permeability [54] are linked to alterations in gut microbiota composition [55]. When there is an imbalance of bacteria in the intestines (bacterial dysbiosis), it can lead to a compromised intestinal barrier, allowing neuroactive compounds from microbes and immune substances to leak into the bloodstream. This leakage has an impact on peripheral inflammation [13].

Current research suggests that the GM plays a significant role in mediating systemic inflammation. This low-grade chronic inflammation has been proposed as a potential factor contributing to chronic pain and other chronic diseases [56]. Numerous studies have also highlighted the established relationship between the GM and immune-mediated inflammatory responses. The GM can modulate the immune response by influencing the function of B- and T-cells, which ultimately affects the body’s resistance to pathogens [57,58,59]. Additionally, it should be noted that the GM in humans produces various hormones or hormone-like compounds, functioning similarly to an endocrine organ. This designation is based on its ability to produce chemicals that can travel through the bloodstream and affect distant sites within the body [60]. The first category consists of substances that regulate pain, such as SCFAs and neurotransmitters. SCFAs are produced by the GM in the large intestine through the fermentation of dietary fibers [61]. Propionate is mainly generated by bacteria from the *Bacteroidetes* phylum, while butyrate is primarily produced by bacteria from the *Firmicutes* phylum [62,63]. SCFAs play a crucial role in controlling intestinal inflammation and maintaining epithelial barrier function. Dysfunction at this level can contribute to “leaky gut syndrome”, which is believed to be implicated in various painful conditions, although it is still not accepted by the medical community as there is little direct evidence regarding this condition [64]. Additionally, circulating levels of SCFAs can influence systemic inflammation modulation as they enter the bloodstream. Notably, individuals with FM showed significant alterations in their serum levels of butyrate and propionate compared to healthy controls [12].

Microbes break down resistant starches and dietary fibers through decomposition and fermentation, producing SCFAs such as formic, pyruvic, butyric, lactic, and acetic acids that are delivered to the host [65]. Along with metabolizing bile acids and other compounds, gut bacteria are believed to be the primary source of SCFAs [66,67]. These SCFAs have been proven to regulate inflammation by recruiting leukocytes, promoting chemokine production, maintaining integrity in the intestinal wall’s tight junctions [68], and safeguarding the blood–brain barrier (BBB) [69]. The composition of the GM also plays a significant role in microglial homeostasis by generating fermentation products derived from microbiota activity [2].

Microglia cells are a vital component of the structural framework of the nervous system, forming connections with astrocytes. They function as neuromodulators, influencing the excitability of central nervous cells and sensory neurons in the spinal cord. Conditions characterized by heightened sensitivity to pain, like FM and CRPS, may be influenced by the activation of microglial cells due to proinflammatory cytokines [23]. Animal models have shown an increase in both activated microglia and their proliferation during acute, inflammatory, and neuropathic pain scenarios [70,71,72,73,74,75,76]. Activated microglia initiate various defense mechanisms, such as clearing toxic debris through phagocytosis, processing antigens for presentation purposes, and releasing multiple cytokines [77,78]. These functions are regulated not only by other glial cells and neurons but also by external factors like SCFAs produced by the gut microbiota. The production of proinflammatory substances, such as TNF-α and IL-1β, contributes to nerve fiber sensitization within the CNS, leading to heightened transmission of pain signals [13].

The connection between microglia–pain, microglia–microbiome, and pain–microbiome suggests that there may be a relationship between intestinal dysbiosis and the activation of microglial cells in the development of chronic pain conditions. Moreover, patients with CRPS exhibit elevated levels of activated microglia in their spinal cord and brain, as well as reduced diversity in gut microbial populations [79,80,81]. Activation of the vagus nerve has been shown to inhibit the proliferation of microglial cells and decrease the expression of proinflammatory cytokines during inflammation caused by lipopolysaccharides. Consequently, signals originating from intestinal microbes may be transmitted through the vagus nerve to influence activation in these immune cells, ultimately contributing to the transmission of pain sensations [13].

The gut–brain axis is a two-way communication system that involves the sympathetic, parasympathetic, and enteric systems. It allows for constant exchange of information between the brain and various bodily functions to maintain balance, including digestion and immune response in the gut [81]. The autonomic nervous system’s efferent nerves play a role in regulating the GI tract and its microbiota by influencing its environment indirectly and directly through signaling molecules like SCFAs, bile acid metabolites, and neuropeptides with antimicrobial properties, such as gamma-aminobutyric acid, tryptophan precursors and metabolites, serotonin, and catecholamines [82]. In addition, the release of cytokines during the immune response to microbes can specifically communicate with host cells in the gut through receptors on local epithelial and mesenchymal cells [82]. These factors can also transmit signals through neurological pathways (such as vagal and possibly spinal afferents) and endocrine mechanisms to target areas beyond the GI tract, including vagal afferents in the portal vein and receptors in the brain [83,84]. While some of these signaling mechanisms can occur even when there is an intact epithelium, they are likely intensified and modified in situations where IP is increased due to stress or mucosal inflammation [85].

Interestingly, the analysis of the CRPS microbiome revealed a decrease in the levels of various bacterial strains that are normally present in a healthy microbiome. These include bacteria associated with the production of SCFAs, such as *Bifidobacterium*, *Eubacterium*, and *Lachnospiraceae* [86,87,88,89]. There is also a reduction in the abundance of *Firmicutes* phylum, indicating dysbiosis patterns among CRPS patients [90]. Dysbiosis can disrupt the intestinal barrier, leading to increased interactions between bacteria and the immune system, which results in localized inflammation [91]. Maintaining an intact intestinal barrier is crucial for SCFA production, particularly butyric acid and butyrate. However, CRPS patients display lower levels of these beneficial compounds due to the decreased presence of certain bacteria like *Eubacterium* species, which play a role in their synthesis. The decrease in the variety of bacteria, particularly those involved in generating beneficial SCFAs, implies that this mechanism could potentially contribute to the onset of CRPS [20].

Extensive evidence suggests the involvement of the immune system in both the onset and persistence of chronic pain. Notably, there are indications that GI bacteria significantly influence immune system function, as individuals with CRPS exhibit lower levels of microbial diversity compared to their healthy counterparts [92,93]. Moreover, decreased diversity in the GI microbiota has been associated with inflammation in the GI tract, impairment of mucosal integrity, and unregulated production of proinflammatory cytokines [94,95,96,97]. It has been proposed that rather than a specific pathogenic bacterium, the cause of GI inflammation is an imbalance in the distribution of healthy gut bacteria [81]. The proximity of these bacteria to the epithelial layer of the GI tract likely plays a role in activating immune cells, which make up 70% of the body’s immune cells within the GI lymphoid tissue. This interaction leads to the production of antimicrobial proteins by epithelial and plasma cells. Previous studies on GI community structures have shown that *Firmicutes* and *Bacteroidetes* are major contributors at the phylum level when considering their relative abundance [98]. The composition of *Firmicutes* and *Proteobacteria* varied significantly between the CRPS and control groups. Notably, individuals with CRPS had higher levels of *Proteobacteria*, which consists mainly of Gram-negative bacteria. Gram-negative bacteria’s cell walls contain lipopolysaccharides, triggering an innate immune response in humans characterized by cytokine production, inflammation, and sickness response. Toll-like receptor 4 (TLR4), a pattern recognition receptor crucial to the innate immune system, plays a key role in recognizing microbial elements associated with inflammation following tissue injury [81].

Moreover, SP levels, which are elevated in IBS, have been implicated in the development of CRPS, particularly among women who are more susceptible to this condition. Furthermore, SIBO-induced increased IP can lead to lipopolysaccharide translocation and activation of microglia activity in CRPS. Evidence indicates that manipulating the microbiome through long-term cephalosporin therapy may be beneficial for managing CRPS symptoms based on reported cases of remission [23].

Additionally, heightened levels of TNF-α and IL-6 proinflammatory cytokines have been observed in correlation with increased IP [99]. The initiation of an immune response in the peripheral region can contribute to inflammation within the CNS, including the activation of microglia [100,101]. Elevated levels of peripheral inflammation are linked to compromised integrity of the BBB and subsequent central inflammation. This compromised BBB allows for small bacterial components and metabolites to enter the CNS, triggering abnormal microglial activity [102]. Disruptions in intestinal barrier integrity have been reported across various chronic pain conditions, such as FM, CRPS, and IBS. The severity of pain and plasma levels of proinflammatory cytokines, such as IL-2, IL-6, and TNF-α, are influenced by the extent of IP [103,104]. These findings suggest that a compromised gut barrier contributes to both systemic and central inflammation and plays a role in the development of chronic pain. The relationship between an impaired intestinal barrier and conditions associated with chronic pain is still not fully understood; it could be either a cause or an effect of the pain response. There is likely a bidirectional mechanism where imbalances in gut bacteria lead to increased IP, which initiates chronic pain. This further disrupts gut homeostasis and alters microbial communities [13].

The composition of intestinal microbes in adults tends to remain stable over time and is unique to each individual. However, various external factors, such as genetics, mode of birth delivery, diet, age, antibiotic treatments, and probiotics, can potentially influence this microbial composition. The intestinal microbiota plays an active role in several processes, including improving nutrient absorption and breaking down non-digestible compounds in the diet [105,106,107,108]. Additionally, it provides essential nutrients while removing harmful toxins and non-nutritional substances. Given the rise of antibiotic-resistant pathogens and the negative impact that antibiotics have on beneficial bacteria, the development of supplementary or alternative therapies based on bacterial replacement is crucial for preserving a healthy microbiota [109].

## 3. Boron: Essential Element in Host–Microbiota Symbiosis

Boron (B) was recently claimed as an essential element for the human host–microbiota healthy symbiosis, and the effects of B deficiency in the microbiota could lead to: (i) dysbiosis, claimed to happen due to deficiency of the autoinducer-2–furanosyl borate diester (AI-2B) signaling molecule; and (ii) *degradation of the mucus gel layer*, due to the lack of B content in the mucin gel structure, which causes the interaction of the bacterial biofilm directly with the host cell membranes and, therefore, their direct infection [110,111,112].

Recent new insights into B’s mechanism of action (MoA) are based on claims that: (i) B is an essential element for the symbiosis between the commensal microorganisms in the microbiome and the human and animal host; (ii) B is not required by the human cell; the human host cells do not need B nutritionally, with this element being necessary only for a healthy symbiosis between the host organism and the various microbiomes of the gut, scalp, mouth, skin and vagina; (iii) some naturally occurring prebiotic boron complexes (PBCs) have recently been proven to be microbiota-accessible boron complexes (MABCs) [110,111,112,113]. More than that, PBCs, such as B-containing pectic polysaccharides (BPPs) and the recently discovered borate complexes of chlorogenic acid (diester chlorogenoborate—DCB), are indigestible compounds [112], while inorganic B compounds, such as boric acid (BA) and borate salts, are digestible and, in certain circumstances, they can be toxic [114,115,116].

These observations helped to formulate a new perspective on the essentiality of B in the animal kingdom, pretending that the AI-2B signaling molecule is actually able to modulate microbiota (composition, bacteria behavior, and community dynamics) as well as the mucus gel layer under conditions of dysbiosis [117]. AI-2B is an emerging signaling molecule that functions as a cross-species and inter-kingdom bacterial communication signal [110]. Furthermore, imbalances in the gut microbiota and increased gut permeability can result from inadequate B levels during symbiosis, leading to a decrease in AI-2B levels [118]. The production of AI-2B by one phylum could influence the gene expression of other species and facilitate interspecies communication, making bacteria change their behavior, especially luminescence, virulence, and formation of biofilm between various species [119]. This characteristic makes AI-2B a highly promising candidate for regulating cell interactions within the human gut and microbiota. It also functions as a widely recognized signaling molecule for coordinating cell behavior across both prokaryotic [120] and eukaryotic species [121]. Recently, the AI-2B molecule has been proposed as a biomarker for dysbiosis [110,112,113]. In addition, B has recently been declared an essential element involved in the synthesis of the autoinducer-2 (AI-2) quorum sensing (QS) system. At the same time, active AI-2B is generated by the addition of borate to the AI-2 precursor, being able to amplify the activity of AI-2 and support the secretion of extracellular polymeric substances [122,123].

Several natural organic boron (NOB) species have been detected in bacteria (borate polyketides, borate–siderophore complexes, AI-2B), fungi (borate esters of carbohydrates), and plants (rhamnogalacturonan II–borate complex (BPP), borate esters of carbohydrates, borate esters of organic acids, *bis*-N-acetyl serine, and borate complexes of phenolic acids, such as DCB) [112,113,124,125]. From digestibility assays of NOBs, only the prebiotic properties of BPP [126,127] and DCB complexes extracted from green coffee beans (GCB) [112] were highlighted. These organic B species are distinct from inorganic BA/borates, which aren’t prebiotic because they are digestible and could be toxic to the microbiota [124,128].

**Figure 1:**
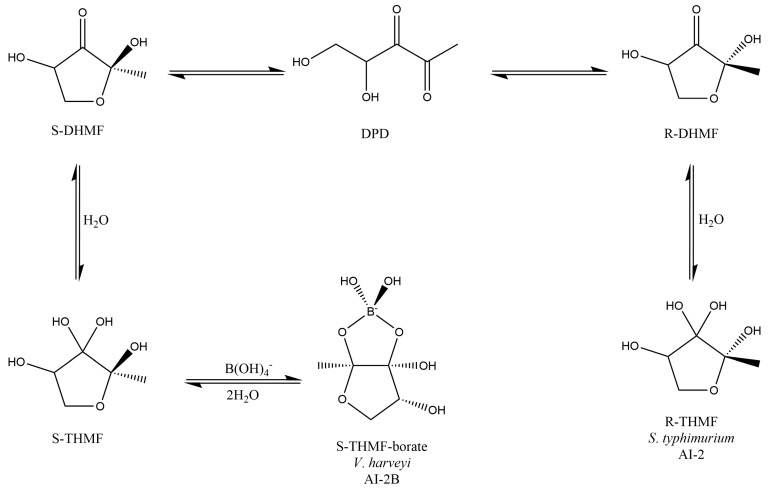
AI-2 pathway: DPD easily cycles into *R*-DHMF and *S*-DHMF stereoisomers. *R*-DHMF and *S*-DHMF are solvated to establish *S*-THMF (AI-2 and AI-2B, two different types of AI-2 signal molecules in water). LuxP receptors connect the cyclic borated form (2*S*,4*S*-THMF-borate), and LsrB receptors connect the non-borated cyclic form (2*R*,4*S*-THMF). AI-2: Autoinducer-2; AI-2B: Autoinducer-2–furanosyl borate diester; DHMF: 2,4-Dihydroxy-2-methyldihydro-3-furanone; DPD: 4,5-Dihydroxy-2,3-pentanedione; THMF: 2-Methyl-2,3,3,4-tetrahydroxytetrahydrofuran. Scheme adapted from Ascenso et al. (2019) [129].

The proposed MoA of B for PBCs is connected to both the fortification of mucus glycoprotein gels with PBCs and the synthesis of AI-2B molecule. Outside the cell, PBCs can react with 4,5-dihydroxy-2,3-pentanedione (DPD) or a hydrate thereof, producing the AI-2B signaling compound. In aqueous solutions, (*S*)-DPD is in balance with its two stereoisomers (*R*-2,4-dihydroxy-2-methyldihydro-3-furanone (*R*-DHMF) and *S*-DHMF) (Figure 1).

For instance, AI-2B participates in intracellular communications with other symbiotic bacteria in the microbiome. It can also react with mucins (high molecular weight glycoproteins) to produce borate-stabilized glycoproteins. These borate-stabilized glycoproteins are integrated by the mucus gel layer of the gut, protecting the integrity of the intestinal wall in the host organism [110,111,112,113].

Researchers have noticed a strong correlation between intestine microbiota and illnesses, such as GI diseases (e.g., IBD, colon cancer, gastroenteropancreatic neuroendocrine neoplasms), cardiovascular diseases (CVDs) (e.g., atherosclerosis), psychiatric diseases (e.g., multiple sclerosis (MS), Alzheimer’s disease (AD), Parkinson’s disease (PD)), metabolic diseases (e.g., obesity and type 2 diabetes (T2D)), respiratory diseases (e.g., chronic obstructive pulmonary disease, bronchial asthma, infectious lung diseases), immune diseases (e.g., rheumatoid arthritis (RA) and ankylosing spondylitis), and so on [98,130]. A main characteristic of the microbiota is the difference between sex, which also means various nutritional needs of B between males and females. Thus, females are more exposed to illnesses caused by prebiotic B deficiency in their nutrition. Supervising the B level and/or the level of AI-2B molecule in feces will show new scientific ways for the illness’s avoidance due to the lack of prebiotic B in nutrition [111,112,113]. The various types of GM in males and females are influenced by sex hormones, which result in different male and female immunities, as well as susceptibility to many infections and chronic diseases [131]. At the same time, the amount of B in male and female’s hair is different since women have almost two times less B in their hair; this means that women need about two times more B in nutrition than men [132]. Several diseases have been explored in relation to the GM and gender differences and the effects of B in nutrition, such as osteoarthritis (OA), osteoporosis (OP), periodontal disease, polycystic ovary syndrome (POS), ovarian cancer, IBD, obesity, fatty liver disease (FLD), T2D, depression, allergic diseases, CVDs, and ischemic stroke [131,133].

The new findings into B essentiality for humans require a new approach to determine B’s status in science. Thus, the MoA of B needs to be identified, therefore explaining the effect of B on human health. Moreover, PBCs are nutritionally essential for healthy human host–microbiota symbiosis, while inorganic B species are not prebiotics [110] since they are digestible.

## 4. Autoinducer-2–Borate Deficiency and Dysbiosis

Recently, PBCs have been claimed to ensure communication between bacteria and the host through AI-2B as well as by B-stabilized mucins in the intestinal mucus [110,112]. Moreover, the connections between host–microbiota symbiosis disturbance, the so-called dysbiosis, and some chronic illnesses are highlighted [134]. Dysbiosis due to B deficiency in nutrition happens when the AI-2B level in microbiota decreases, the balance between commensal and pathogenic bacteria is broken, and, subsequently, the mucus gel is also destroyed [135].

B is an essential microelement for the synthesis of AI-2 signaling molecules and influences bacterial general behavior. The health of the microbiota was positively correlated with a high concentration of B in the excreted feces [113]. Thus, the level of B in the feces is not correlated with the levels of B in the body of the host organism; rather, the level of B in feces is correlated with a level of B in the gut microbiota and mucin gel layer. High levels of B in feces after administration of non-digestible PBCs indicate a healthy human host–microbiota symbiosis. When the B intake is digestible, e.g., BA or inorganic borates, then the level of B in urine and blood is correlated with increased levels of B in the host organism, for example, an animal or a human, and can become potentially toxic to the host [110].

The MoA of PBCs is as follows: PBC intake (i.e., DCB and BPP) allows bacterial species that communicate using AI-2 to accumulate B in the form of AI-2B, which transfers the borate anion to glycoprotein mucins to fortify the layer of colonic mucus, helping host protection against bacterial infection. The study examined the binding of borate anions to mucins, revealing an observed viscosity increase. This viscosity boost results from the specific binding of borate to diols, thereby augmenting the anionic charge of the polymer [136]. Furthermore, existing mucin separation techniques have also shed light on the interaction between borate and glycosylation sites within mucins [137]. After PBC intake, the amount of B accumulated in the gut mucus gel layer could lead to important effects on human health: boost of AI-2B level, reduction of gut damage, and microbial flora modifications in diarrhea. Thus, many factors lead to microbiota dysbiosis, out of which the most important is the lowering of the AI-2B level, resulting in diminishing the QS system’s ability to maintain the stability of the gut microbiota, thus leading to diarrhea [111,112,113].

Some recent animal and human experiments were carried out regarding the supplementation of PBCs standardized as DCB-rich GCB natural extract, and the level of AI-2B was mainly monitored [113]. Dietary supplementation with DCB improved the diarrhea index and increased AI-2B in a rat model of castor oil-induced diarrhea. In vivo feeding with DCB-rich natural extract was performed on three groups of six-month-old male Wistar rats, with four animals in each group and medium weight of 290 ± 10 g, as follows: Group 1 (control)—rats fed with normal diet; Group 2 (castor oil-induced diarrhea)—rats with castor oil-induced diarrhea; Group 3 (DCB)—rats fed with DCB-rich GCB natural extract (as 15 ppm B) five days after the onset of diarrhea. During the study, the rats were put in individual cages and monitored under standard conditions of humidity, lighting, and temperature (12 h light/dark cycle). AI-2B values in induced diarrhea for Group 2 are noted to be very low at an average of 1.4 μM, which corresponds to an increased diarrhea index. The DCB-rich GCB natural extract diet five days after the onset of diarrhea results in a consistent fecal AI-2B concentration of 45.3 μM and a near-normal diarrhea index [113].

The dysbiosis index (DI) in the stool of the patients with long-term antibiotic therapy supplemented with a DCB-rich GCB natural extract was also described [113]. Manipulation of QS AI-2 signaling affects antibiotic-treated gut microbiota. These discoveries provide a new way for the AI-2 to work as a signal molecule for intraspecies and interspecies communication, as increased AI-2 counteracts antibiotic-induced dysbiosis. DCB or a DCB-rich natural extract may serve as a nutritional adjuvant for the gut microbiota in patients undergoing long-term antibiotic therapy for respiratory tract infections, including tuberculosis. The use of DCB-rich natural extract in diarrhea relief increases fecal AI-2B levels and reduces dysbiosis. Group 1: 10 healthy subjects (control); Group 2: 10 subjects with standard intravenous antibiotic treatment (Sulcef); Group 3: 10 subjects with standard intravenous antibiotic treatment supplemented with standardized DCB-rich GCB natural extract (three capsules/day—1 mg B, approx. 65 mg DCB) during the 30 days. For the pilot study, DCB was prepared as GCB natural extract (ca. 6.5% DCB) at a standard concentration of 1000 ppm B. The DI of Group 2, treated with antibiotics, shows a strong increase, and there is a corresponding decrease in the level of AI-2B, which means that the commensal bacteria are reduced in number. Group 3, supplemented with DCB-rich GCB natural extract, shows a reduction in DI compared to Group 2 and a significant increase in the level of AI-2B. Supplementation with natural extracts rich in DCB can prevent dysbiosis induced by antibiotic treatment by increasing AI-2B in the gut microbiota. AI-2B is being proposed by the authors as a potential marker of intestinal dysbiosis [113].

Subsequently, dysbiosis due to the deficiency of PBCs and implicitly of AI-2B determines the disruption of communication between bacteria, between bacteria and the host, and, consequently, the degradation of the mucus gel. Recent results claim that cross-kingdom communication happens between bacteria and eukaryotic cells through the bacterial AI-2 QS system. By raising AI-2 levels, Thompson et al. (2015) noticed that the evolution of *Firmicutes* increased while the development of *Bacteroidetes* stopped, thus inverting the antibiotic-induced dysbiosis. The *Firmicutes* group, which are producers of AI-2, diminishes in abundance following antibiotic treatment, while the *Bacteroidetes* phylum augments in abundance [117,138]. More recently, in research on murine models with neonatal necrotizing enterocolitis (NEC), the concentration of AI-2 was found to have been highly reduced during the acute disease phase but gradually raised in the remission phase [117]. These observations prove that AI-2B and AI-2 have an impact on the balance and structure of the gut microbiota. The connection between AI-2B and intestine microbiota equilibrium shows that AI-2B can be a new biomarker to identify intestine homeostasis [138]. Recent data notice that high levels of AI-2 are thus able to rebuild the equilibrium between *Firmicutes* and *Bacteroidetes* and hinder or invert dysbiosis, obesity, autism, IBD, and disorders connected to aging and stress. These findings that prebiotic B boosts the activity of AI-2 through the ingestion of PBCs and the formation of AI-2B can be used to restore the equilibria in the GM after antibiotic treatment and, in addition, can be a nutritional adjuvant for a long and healthy life [139]. It is known that in children and the elderly, the *Firmicutes*/*Bacteroidetes* ratio is low, while for adults, this ratio increases [140]; aging is an inducing factor of dysbiosis. Subsequently, the health condition of the microbiota symbiosis is important in increasing the healthy and long life of human hosts and increasing stress resistance. Research has proved that aging is correlated with many modifications, such as decreases in GM variation, decreases in the rates of *Firmicutes* and *Bacteroidetes*, abundances of pathogens, and decreases of bacteria producing the SCFAs necessary to maintain composition integrity and stop inflammation in the intestine [52,65,141,142,143].

Non-digestible carbohydrates dietary fibers are used as prebiotics, but recently, polyphenols can also function as prebiotics to generate butyrate. Intake of polyphenols increases the abundance of butyrate-bacteria producers, such as *Faecalibacterium* and members of the *Ruminococcaceae* family. Other phenolic compounds, such as caffeic acid, chlorogenic acid, and rutin, are also reported to boost microbial butyrate [144,145,146].

Deficiency of PBCs in the diet correlates with the reduction of AI-2B levels in the microbiota with direct consequences on *Firmicutes*, which are the majority producers of the butyrate, with direct effects on healthy human host–microbiota symbiosis. Recent evidence shows that dysbiosis is linked to low production of butyrate [147]. The effects of prebiotic B deficiency are translated by the decrease of *Firmicutes*, the decrease of AI-2, and, implicitly, of AI-2B. Over 200 species of bacteria from the *Firmicutes* species make a major contribution to the commensal microbiota. B compounds accessible to the microbiota increase the level of acetate, especially butyrate, with effects on the brain, bones, and the immune system of the intestine. The lack of PBCs will be felt through dysbiosis, mucus degradation, and, implicitly, the decrease in the level of butyrate in the microbiota, and this will lead to a pleiotropic effect on the skeletal system (gut–bone and gut–cartilage axes) and the immune system (inflammation of the cardiovascular system, brain, autoimmune pathologies) [148].

Moreover, the lack of PBCs in food favors pathogenic bacteria and causes dysbiosis and degradation of the intestinal mucus, with consequences on the health of the host through the appearance of both local and systemic diseases (GI diseases, inflammation of the cardiovascular system, and brain and skeletal system degradation), and in the end, even breaking the cycle of life [110]. A molecule is considered to be an essential nutrient if the diet deficiency results in biological dysfunction. Dysbiosis can influence the function of the gut barrier by changing the thickness of the mucus, and this leads to the progression of many illnesses. Predominantly, bacteria that reside within humans are either commensal or mutual, and the mucus layer is the first line of defense against the penetration of microbiota, acids and digestive enzymes, digested food components, and food-associated toxins. Mucus is thought to serve as a source of nutrients for commensal microbiota [149,150].

Therefore, the presence of B in the microbiota through the diet with PBCs has the following MoA: (i) increasing the level of AI-2B that prevents pathogenic bacteria and stimulates the growth of commensal bacteria, especially Firmicutes [111,112,138]; (ii) improving dysbiosis and strengthening the mucus gel of the gut by increasing B content in the mucin gel structure [110]; (iii) increasing the level of *Firmicutes* and other AI-2-dependent bacteria (*Clostridium*, *Coprococcus*, *Eubacterium*, *Ruminococcus*) [151]; (iv) increasing the total amount of SCFAs, especially butyrate; (v) increasing B in colon; butyrate-producing colonic microbiota stimulate 1,25-dihydroxyvitamin D (vitamin D3) production by colon-resident immune cells [152,153].

## 5. Perspectives to Use the Prebiotic Boron-Containing Compounds in Complex Regional Pain Syndrome

MABCs hold promise as a potential therapeutic approach in CRPS, a debilitating chronic pain condition that affects a considerable number of individuals worldwide. Several studies have highlighted the role of the gut microbiota in the modulation of pain responses and the development of chronic pain conditions, such as FM and IBS.

Recent research has shown that alterations in gut microbiota composition and IP are associated with chronic pain conditions. This suggests that targeting the microbiota–gut–brain axis could be a potential path for therapeutic intervention in CRPS. MABCs have emerged as a potential therapeutic strategy due to their ability to modulate gut microbiota composition and restore gut homeostasis. These compounds have been shown to improve IP and reduce inflammation, which are key factors in the development of chronic pain.

In our opinion, prebiotic B deficiency in the diet causes dysbiosis and degradation of the gel mucus in the intestinal tract. Damage in the gut mucus layer aggravates gut inflammation and infection. The intestine microbiota sends bidirectional signals to organs, affecting the metabolism of the whole body and is a contributing factor to metabolic illness. A damaged gut mucosal barrier can be an essential interface between the host and flora. PBCs also improve immunity, have antioxidant and anti-inflammatory actions on the microbiome [154], and determine the health of the body through the main “axes” defined in the scientific literature: “microbiota–gut–brain axis”, “microbiota–gut–bone axis”, “microbiota–gut–thyroid axis”, “microbiota–gut–cartilage axis”, and “microbiota–gut–heart axis” [155]. Subsequently, PBCs have a major role in the avoidance of certain illnesses, such as OA, OP, RA, cardiovascular inflammation, depression, obesity, T2D, and thyroid diseases [156].

The indigestible PBC species are microbiota-accessible and cause an increase in the level of volatile fatty acids due to the increase in the activity of commensal bacteria, especially the level of butyrate producers [157]. Most human butyrate producers belong to the *Firmicutes* phylum, including species such as *Butyrivibrio fibrisolvens*, *Clostridium butyricum*, *C. kluyveri*, *Eubacterium limosum*, and *Faecalibacterium prausnitzii* [158]. In addition, other bacteria produce butyrate; *Anaerostipes* spp., *Bifidobacterium* spp., and *E. hallii*. *Bifidobacterium* spp. generate butyrate from lactate (a product of glucose metabolism) and acetate. All primary butyrate-producing bacteria are anaerobic, meaning they can only grow in low-oxygen environments. Because oxygen levels in a healthy colon are extremely low, these organisms thrive in this ecosystem. The *Firmicutes* phylum has recently been shown to increase intestinal levels of AI-2 and butyrate. Acetate and propionate are the main products of the *Bacteroidetes* phylum. Thus, the increase in AI-2 levels favored the expansion of *Firmicutes* in the microbiota treated with antibiotics and inhibited *Bacteroidetes*, therefore counteracting the dysbiosis induced by antibiotic treatment [117,159].

**Figure 2:**
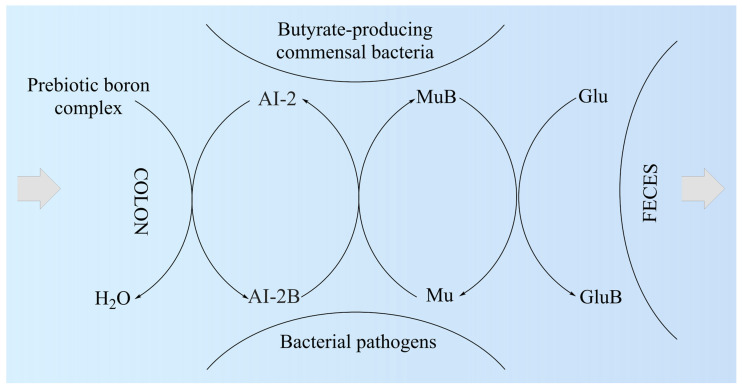
The proposed mechanism of action of prebiotic boron complex as a mediator for AI-2B and MuB production. AI-2: Autoinducer-2; AI-2B: Autoinducer-2–furanosyl borate diester; Glu: Monosaccharide (mainly fucose and sialic acid); GluB: Monosaccharide–boron complex; Mu: Mucin gel; MuB: Mucin gel–borate complex.

We proposed the MoA of PBCs as a mediator for the production of butyrate in the colon (Figure 2). Thus, the AI-2B level increases and stimulates the growth of butyrate-producing bacteria (mainly *Firmicutes*) [151]. AI-2B transfers the borate anion to mucins stimulated by butyrate and, through the high level of AI-2B, inhibits pathogenic bacteria (such as *Vibrio* spp.) [160,161,162]. The reverse process of decreasing prebiotic borate intake leads to the inhibition of butyrate-producing bacteria and the growth of pathogenic bacteria that are stimulated by low AI-2B. In addition, the lack of PBCs in the diet accelerates the loss of B from the mucus in the feces by complexing, mainly with fucose and sialic acid (Figure 2). Subsequently, PBCs are the ones that have an essential impact on host–microbial symbiosis in health, and mainly on butyrate-producing bacteria and AI-2 signaling molecules, according to the model shown in Figure 2, in two directions: microbiota–gut–immune system and microbiota–gut–musculoskeletal system.

Butyrate is known to be a key metabolite of the GM that mediates the effects on the immune system cells (T-cells, antigen-presenting cells, monocytes, and neutrophils) and plays a key function in maintaining gut immune homeostasis, but also has potential future therapeutic for a spectrum of intestine and systemic illness [148,163,164,165,166,167]. Recent studies have found that butyrate-producing microbes protect against or are associated with less severe symptoms of a long list of conditions related to chronic inflammation: allergies, IBS, RA, PD, high blood pressure, insomnia, anxiety, and T2D [148]. Moreover, butyrate is involved in boosting the mechanical and immune barrier of the intestine and promotes a healthy intestinal barrier, preventing the “leaky gut” syndrome [168], and has been reported to be an inhibitor of histone deacetylases (HDACs). A growing number of studies have found that butyrate may exert protective effects on atherosclerosis, hypertension, and vascular health [148].

Subsequently, butyrate-producing bacteria of *Firmicutes* filum can have a key role in the healthy symbiosis of the human colon as a major energy source for the colonic mucosa, increasing mucin secretion, and as an important modulator of gene expression, inflammation, differentiation, and apoptosis in host cells with a function in improving intestinal barrier function [169,170]. B stimulates butyrate-producing bacteria through AI-2B. Butyrate stimulates the de novo synthesis of mucins, ensures the transfer of B to them, and catalyzes the incorporation of B-stabilized mucins into mucus (Figure 2). Mucins are the growth substrate for butyrate-producing *Firmicutes*, and because mucosal butyrate producers release butyrate close to the epithelium, they may increase the bioavailability of butyrate to the host [171].

*Firmicutes* are the main butyrate-producing bacteria in the human colon, especially *E. rectale*, *Clostridium leptum*, *Roseburia* spp., and *F. prausnitzii* [172,173]. Furthermore, butyrate plays a role in controlling the synthesis of cathelicidins, which are polycationic peptides involved in the innate immune system of mammals and demonstrate wide-ranging antimicrobial capabilities against potential intestinal pathogens. Conversely, a decreased presence of butyrate-producing organisms in the intestinal environment leads to the proliferation of aerobic bacteria from the *Enterobacteriaceae* family, which is commonly associated with signs of intestinal dysbiosis [174].

**Figure 3:**
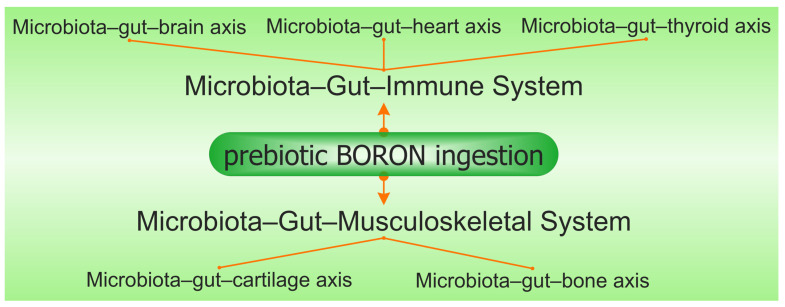
The synergy involving prebiotic boron, the microbiota–gut–immune system, and the microbiota–gut–musculoskeletal system is mediated via the microbiota–gut–organ axis.

The number of articles which study the in vitro and in vivo effects of B on butyrate and SCFA production is limited. There are a few studies that show that B as a feed additive can improve ruminal microbial fermentation and promote SCFA formation, especially an increase in butyrate by 40%, while total SCFA increases by over 60% [157,175]. A recent study proved that B improved the disordered gut morphology, and combined treatments with probiotics reduced both oxidative damage and inflammatory processes [176]. The latest studies have noticed that there are some close correlations between B intake, AI-2, the *Firmicutes* phylum, and the production of butyrate [138,177]. In addition, chlorogenic acid, the ligand in the DCB complex, and other phenolic compounds, such as caffeic acid and rutin, are also reported to increase microbial butyrate [144]. Therefore, DCB, like PBCs, has in its composition both a prebiotic part of phenolic acid and B as an essential nutritional element for butyrate-producing bacteria [112]. Thus, many of the functions of butyrate on health status are also found in the case of prebiotic B ingestion in both animals and humans (Figure 3).

## 6. Microbiota–Gut–Immune System

The immune system is a complex network of biochemical molecules, cells, organs, and tissues that coordinate with each other to fight off invaders and repair damage [178,179,180]. The cooperation between the gut microbiota and the immune system can take place on several main axes:

(i) *Microbiota–gut–brain axis*: There is an expanding interest in the relationship between the microbiome, intestine, and brain, which is defined as the “microbiota–gut–brain axis” [181]. The microbiota–gut–brain axis plays an important role not only in the health of the GI tract but also in brain function and behavior and is modulated at different levels by the enteric nervous system, CNS, hypothalamic–pituitary–adrenal axis, and immune system through a complex communication of immune, endocrine, and neuronal factors [182,183]. In the context of the microbiota–gut–brain axis, a leaky gut has become an important point of interest in neurological disorders in the last decade, with the demonstration of gut microbiota dysbiosis and/or an impaired gut barrier in neurological diseases of various etiologies, such as accident stroke, Huntington’s disease, and amyotrophic lateral sclerosis (ALS). Disruption of the intestinal barrier has also been linked to cognitive impairment associated, or not, with neurodegeneration, and gut microbiota dysbiosis is functionally linked to brain immunological dysfunctions, contributing to poor mental health, neuroinflammation by increasing bacterial metabolites and inflammatory cytokines in the intestine and BBB, and the development of numerous neurodegenerative diseases, including AD, PD, MS, and ALS [184].

The latest research showed that B is a beneficial trace element for some metabolic pathways in the body as many deficiencies of B disturb memory, anxiety level, cognitive functions, mood, and sleep, as well as modulate calcium and magnesium exchange and vitamin D and sex steroid metabolism. B can help create the intestinal organizational structure, which improves GI absorption [185,186,187]. Recently, exposure to B nitride nanotubes was shown to stimulate tadpole growth and was also connected with intestine microbiota remodeling, clearly demonstrating the role of B in modulating microbiome physiology. Nutritional prebiotic B intakes have been noticed to have favorable benefits on central neurological function, with the potential to treat neurological diseases such as PD and AD. At the same time, a B-based diet significantly improves brain function and cognitive functioning in humans and will play a significant role in improving dysbiosis and opening new directions for researchers for perspectives on B nutrition [188,189]. The most impressive feature in the microbiota of the centenarian people is a change in the ratio of *Firmicutes* and *Bacteroidetes*, with old people having a higher proportion of *Bacteroidetes*, while young adults have higher proportions of *Firmicutes* (butyrate-producing bacteria), which are stimulated by PBCs [141]. Butyrate is known to contribute to the modulation of brain-derived neurotrophic factor (BDNF), and it is well known that gut dysbiosis causes decreased BDNF levels, which could affect neuronal development and synaptic plasticity. The use of butyrate-producing bacteria increases attention as and in the conditions in which the PBCs ensure the simulation of butyrate-producing bacteria (*Firmicutes* and *Actinomycetae*), and MABCs can be a promising adjuvant nutritional therapeutic for depression. Aging is associated with low levels of BDNF, suggesting that maintaining adequate levels of BDNF could help prevent or delay the onset of cognitive decline. A convenient way to increase BDNF levels is by supplementing with PBCs, which are SCFAs that function as HDAC inhibitors [190]. This improves brain plasticity, leading to new neural connections and growth and increased cell survival [191,192]. It has recently been shown that adequate B supplementation can support the brain development of ostrich chicks by boosting the BDNF expression and lowering the cell apoptosis process, but a high dose of B intake damages the neuron structure of chick ostrich brain by inhibiting BDNF expression and increasing cell apoptosis [193]. Moreover, an in vitro approach has shown that B-containing compounds could be neuroprotective agents for the management of AD [194]. The MoA of B neuroprotection could relate to: (a) reversed dysbiosis and stopping of degradation of mucus by B-stabilized mucins by AI-2B; (b) the regulation of BDNF and acetylcholinesterase expression levels by gut microbiota-derived metabolite butyrate induced by PBCs [113,195].

(ii) *Microbiota–gut–heart axis*: CVDs are illnesses that affect the blood vessels and heart, i.e., hypertension, heart failure (HF), atherosclerosis, and stroke. Numerous in vitro and in vivo studies proved the cardiovascular protective capabilities of butyrate, especially the protective effects in atherosclerosis, i.e., higher stroke risk relates to a reduced level of butyrate-producing bacteria in the intestine and reduced fecal butyrate concentrations [147,196,197]. Recent results show that B had a positive impact on myocardial infarction-induced HF, attenuated apoptosis, and cardiac fibrosis and has the potential to induce cardiac tissue regeneration after injury [128,198,199].

(iii) *Microbiota–gut–thyroid axis*: Butyrate increases the expression of thyroid hormone receptors in mice, being a well-recognized inhibitor of HDAC. Thus, butyrate increases iodine uptake and the expression of thyroid hormone receptors in mice [200,201]. Regarding the nutritional intake of B, the most important research studies regarding the supplementation of the diet with B in animals and humans have shown the following: (a) healthy women fed with a diet rich in B (10 mg B per day) have a significant decrease of 25% in the thyroid-stimulating hormone (TSH, thyrotropin) level [202]; (b) B shows an iodine-like effect while investigating the influence on tail maturation of *Xenopus laevis* frogs, i.e., tail maturation in *X. laevis* is modulated by the thyroid axis [203,204]; (c) dietary supplementation with BA (400 mg/kg) during a 4-month period increased the level of triiodothyronine (T3) of the rams in the serum samples compared with control [205]. There were also some contradictory results, but in general, favorable effects were observed with the B diet in animals and humans [206]. However, B has a great beneficial role in increasing the secretory activity of the thyroid gland and, recently, on finding that commensal microbes function as T3 deposits, may prevent thyroid hormone fluctuation and, thus, may be able to reduce the need for thyroxine (T4) supplementation. Moreover, B intake inhibits TSH by dopamine (DA)-producing bacteria [207]. How the gut causes thyroid disease may be explained by damage to the gut barrier due to dysbiosis and the subsequent increase in gut permeability, allowing antigens to pass through more easily and activate the immune system or cross-react with extraintestinal tissues, respectively [208]. The lack of PBCs in food determines B deficiency in the large intestine, that is, where 90% of the commensal bacteria are, and dysbiosis and implicit cellular permeabilization at the colon level with devastating effects for the human body. B ensures a healthy symbiosis and is involved in the storage of T3 hormone in the microbiota, and with direct action on the DA hormone, controls thyroid function. DA is a physiological regulator of TSH secretion. There are recent data that demonstrate the link between the level of DA and the gut microbiota-derived metabolite butyrate, with DA having a regulatory effect on the synthesis of colonic mucus [209]. Therefore, the presence of B in the microbiota through the diet with MABCs has the following MoA: (i) B increases the level of AI-2B; (ii) it improves dysbiosis and strengthens the mucus gel of the cell wall; (iii) it increases the level of butyrate, which causes an increase in DA and reduces the level of TSH, with major implications on the metabolism of iodine and, therefore, T3 hormone [210]; (iv) B increases T3 deposits from the microbiota, with increases in the level of AI-2B and butyrate [113,187,211].

## 7. Microbiota–Gut–Musculoskeletal System

The musculoskeletal system comprises muscles, bones, tendons, ligaments, cartilage, and other connective tissues, and its primary roles include providing structural support to the body, safeguarding vital organs, and enabling bodily movement [212]. The relation between the GM and the musculoskeletal system can take place on two main axes: microbiota–gut–cartilage and microbiota–gut–bone.

(i) *Microbiota–gut–cartilage axis*: In the research paper entitled “Essentiality of boron for healthy bones and joints”, Newnham wrote that “in areas of the world where boron intake is 1.0 mg or less/day, the incidence of arthritis varies from 20 to 70%, while that in areas where boron intake is 3 to 10 mg, the incidence of arthritis ranges from 0 to 10%” and B levels in sera was lower in patients with OA [213,214,215]. A recent approach showed that oral and intraperitoneal injection of BA is beneficial for rats with induced OA. The effect of BA on cartilage lesions was investigated and showed that the healing process of cartilage lesions could be improved by injecting BA into the knee joint of rabbits [216,217]. Moreover, it has been proved that B showed significant improvement in knee OA symptoms and reduced blood levels of C-reactive protein in patients suffering from OA [124,218]. In recent years, emerging evidence has indicated that dysbiosis and changes in butyrate metabolite production are closely related to OA pathogenesis. These findings provide insight into potential strategies targeting butyrate-producing bacteria for the prevention and treatment of OA [209]. Butyrate reduces pain caused by OA, has chondroprotective effects, and reduces articular cartilage degeneration [219,220]. Butyrate modulates levels of inflammatory mediators, anabolic factors, and catabolic factors in OA. These findings provide insight into potential strategies targeting the stimulation of butyrate-producing bacteria by PBCs and reversing dysbiosis and permeabilization stopping by B-stabilized mucins for the prevention and treatment of OA [220].

(ii) *Microbiota–gut–bone axis*: Within the microbiota–gut–bone axis, butyrate promotes osteoblast differentiation and stimulates the formation of mineralized nodules to support bone growth and has been reported to directly stimulate osteoblast activation and bone formation, promote calcium absorption, and significantly improve bone strength and bone mineral density [221,222,223,224]. Furthermore, elevated levels of free fucose and sialic acid have been documented during antibiotic therapy. This could be attributed, at least in part, to the heightened presence of mucin-degrading *Bacteroides*, which has been observed to proliferate following antibiotic treatment [225,226]. In short, butyrate has a positive effect on the maintenance of bone health by regulating the immune function of the animal body and mitigating bone destruction and loss, and, as a result, the beneficial effects of B on bone health can be correlated with a healthy symbiosis of the *Firmicutes* phylum, the largest producer of butyrate [147,168,227,228]. B helps the body use vitamin D more efficiently, acting as a stabilizer for this otherwise ephemeral nutrient. It is not just the advantages that B provides to bone health; it also plays a crucial role in maintaining hormone balance, supporting brain health, and exhibiting anti-inflammatory properties. Nevertheless, numerous human health studies indicate that the beneficial effects of B are noticeable when the daily dose equals or exceeds 3 mg. When it comes to incorporating B into your diet, like many other essential nutrients, the key is to consume a variety of fresh, whole fruits, vegetables, and nuts. Foods such as avocados, apricots, cashews, almonds, dates, peanuts, prunes, raisins, lentils, and hazelnuts are prime examples of vitamin B-rich foods and rank among the finest sources of dietary prebiotic B [229]. Excessive alcohol intake, heightened stress, heavy smoking, and a family background of OP are factors associated with elevated urinary B levels. This increase in B excretion could potentially lead to greater bone deterioration, especially in postmenopausal individuals. Among females and males over 40, urinary levels of cortisol, adrenaline, and DA were found to be elevated and correlated with increased calcium excretion. This implies that the high levels of catecholamines in these individuals may contribute to calcium loss and potentially compromise bone health as they age [230]. Subsequently, for the microbiota–gut–bone axis, nutritional adjuvant PBCs stimulate bone formation by microbial metabolite butyrate and may represent a therapeutic strategy to enhance bone anabolism [231].

## 8. Conclusions

The intricate relationship between the GM and neuropathic pain in CRPS reveals a fascinating and potentially pivotal aspect of this debilitating condition. The growing body of research exploring how gut dysbiosis and alterations in the microbiota–gut–brain axis may contribute to neuropathic pain underscores the need for further investigation. While much remains to be understood about the precise mechanisms and causal links involved, these findings hold significant promise for the development of novel therapeutic strategies targeting the GM to alleviate neuropathic pain in CRPS patients. As we delve deeper into this complex interplay, we open new paths for potential relief and improved QoL for those affected by CRPS, offering hope for more effective treatments in the future.

B is a crucial element for improving the symbiotic relationships between commensal microorganisms in the microbiome and their human or animal hosts. It is important to note that human or animal host cells do not have a nutritional requirement for B themselves. Instead, B is essential solely for maintaining a healthy symbiotic relationship between the human host and the diverse microbiomes found in the gut, scalp, mouth, skin, and vagina.

In the future, butyrate and AI-2B have the potential to serve as markers for detecting gut dysbiosis. The consumption of PBCs (such as BPPs or DCB) found in fruits and vegetables can help restore the balance between *Firmicutes* and *Bacteroidetes* and prevent or reverse dysbiosis. There is a significant link between nutrition rich in PBCs, AI-2B, the *Firmicutes* phylum, and other butyrate-producing bacteria. A deficiency of these prebiotic compounds in the diet can lead to dysbiosis and the breakdown of the mucus barrier in the intestinal tract.

The lack of prebiotic B will be felt through dysbiosis, the degradation of mucus, and, implicitly, the decrease in the level of butyrate and AI-2B in the feces, and this will lead to a pleiotropic effect on the skeletal system (gut–bone and gut–cartilage axes) and the immune system (inflammation of the cardiovascular system, brain, autoimmune pathologies—MS, thyroiditis, etc.).

An essential feature of the microbiome is the difference between the sexes, which also translates into different nutritional requirements for B between the sexes. Several diseases have been explored in relation to the GM and gender differences and the effects of B in nutrition, such as OA, OP, periodontal disease, ovarian cancer, POS, obesity, IBD, T2D, FLD, allergic diseases, depression, ischemic stroke, and CVDs.

Consequently, evidence suggests that B may hold promise as a potential therapeutic pathway for individuals suffering from CRPS. While further research is needed to establish the precise mechanisms of B’s action and its optimal dosage for CRPS management, the potential benefits it offers in terms of pain relief, anti-inflammatory activity, and overall health support make it an intriguing area for future investigation. As science continues to unravel the complexities of CRPS, B remains a noteworthy candidate for inclusion in the arsenal of treatments aimed at improving the QoL for those afflicted by this challenging condition.

## Data Availability

Data supporting reported results can be found at: https://docs.google.com/document/d/1FB0A3i2aY1EnL3hTdXJeIkdGNGmE0Ak0/edit?usp=sharing&ouid=103898018898457516549&rtpof=true&sd=true (accessed on 23 September 2023).
